# [2-(Phenyl­diazen­yl)pyrrolato]bis(2-pyridylphen­yl)iridium(III)

**DOI:** 10.1107/S1600536808004443

**Published:** 2008-02-20

**Authors:** Wen-Ying Li, Li-Sheng Mao, Long Lu, Hong-Wu He

**Affiliations:** a Key Laboratory of Pesticides and Chemical Biology of the Ministry of Education, College of Chemistry, Central China Normal University, Wuhan 430079, People’s Republic of China; b Shanghai Institute of Organic Chemistry, Chinese Academy of Sciences, Shanghai 200032, People’s Republic of China

## Abstract

In the title compound, [Ir(C_10_H_8_N_3_)(C_11_H_8_N)_2_], the Ir center is octa­hedrally coordinated by the three chelating ligands, with two cyclo­metalated 2-pyridylphenyl ligands [Ir—N = 2.049 (5) and 2.030 (5) Å; Ir—C = 2.016 (6) and 2.012 (6) Å] and a bidentate 2-(phenyl­diazen­yl)pyrrolate ligand [Ir—N = 2.204 (5) and 2.079 (5) Å]. The Ir—N(diazen­yl) bond is longer than the Ir—N(pyrrolate) bond. The structure is stabilized by aromatic π–π stacking, the shortest parallel distance between ring centroids being 3.426 (8) Å..

## Related literature

For phospho­rescence properties of cyclo­metalated iridium complexes, see: Baldo *et al.* (2000 ▶); Pomestcheako *et al.* (2003 ▶); Chen *et al.* (2003 ▶). For the preparation of iridium complexes, see: Lamansky *et al.* (2001 ▶); Davies *et al.* (2006 ▶). For reference structural data, see: Allen (2002 ▶); Allen *et al.* (1987 ▶); Chin *et al.* (1995 ▶).
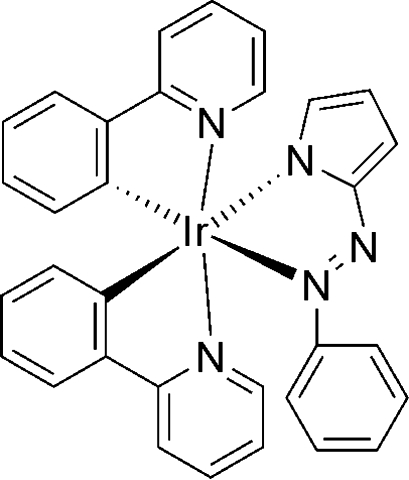


## Experimental

### 

#### Crystal data

[Ir(C_10_H_8_N_3_)(C_11_H_8_N)_2_]
*M* *_r_* = 670.76
Monoclinic, 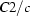

*a* = 17.5606 (14) Å
*b* = 11.0213 (9) Å
*c* = 26.673 (2) Å
β = 93.282 (1)°
*V* = 5153.9 (7) Å^3^
*Z* = 8
Mo *K*α radiation
μ = 5.21 mm^−1^
*T* = 293 (2) K
0.22 × 0.14 × 0.06 mm

#### Data collection

Bruker SMART APEX CCD area-detector diffractometer
Absorption correction: multi-scan (*SADABS*; Sheldrick, 2001 ▶) *T*_min_ = 0.754, *T*_max_ = 1.000 (expected range = 0.549–0.728)
14220 measured reflections
5327 independent reflections
4228 reflections with *I* > 2σ(*I*)
*R*_int_ = 0.140

#### Refinement

*R*[*F* ^2^ > 2σ(*F* ^2^)] = 0.048
*wR*(*F* ^2^) = 0.107
*S* = 0.97
5327 reflections
343 parameters
H-atom parameters constrained
Δρ_max_ = 4.15 e Å^−3^
Δρ_min_ = −2.38 e Å^−3^

### 

Data collection: *SMART* (Bruker, 2001 ▶); cell refinement: *SAINT-Plus* (Bruker, 2001 ▶); data reduction: *SAINT-Plus*; program(s) used to solve structure: *SHELXS97* (Sheldrick, 2008 ▶); program(s) used to refine structure: *SHELXL97* (Sheldrick, 2008 ▶); molecular graphics: *SHELXTL* (Sheldrick, 2008 ▶); software used to prepare material for publication: *SHELXTL*.

## Supplementary Material

Crystal structure: contains datablocks I, global. DOI: 10.1107/S1600536808004443/at2539sup1.cif

Structure factors: contains datablocks I. DOI: 10.1107/S1600536808004443/at2539Isup2.hkl

Additional supplementary materials:  crystallographic information; 3D view; checkCIF report

## Figures and Tables

**Table:** 

Ir—C22	2.012 (6)
Ir—C21	2.016 (6)
Ir—N5	2.030 (5)
Ir—N4	2.049 (5)
Ir—N3	2.079 (5)
Ir—N1	2.204 (5)

**Table:** 

C22—Ir—C21	85.1 (2)
C22—Ir—N5	79.7 (2)
C21—Ir—N5	96.0 (2)
C22—Ir—N4	94.9 (2)
C21—Ir—N4	79.4 (2)
N5—Ir—N4	173.2 (2)
C22—Ir—N3	96.8 (2)
C21—Ir—N3	174.5 (2)
N5—Ir—N3	89.5 (2)
N4—Ir—N3	95.3 (2)
C22—Ir—N1	170.6 (2)
C21—Ir—N1	104.0 (2)
N5—Ir—N1	97.2 (2)
N4—Ir—N1	88.74 (19)
N3—Ir—N1	74.2 (2)

**Fig. 1.**
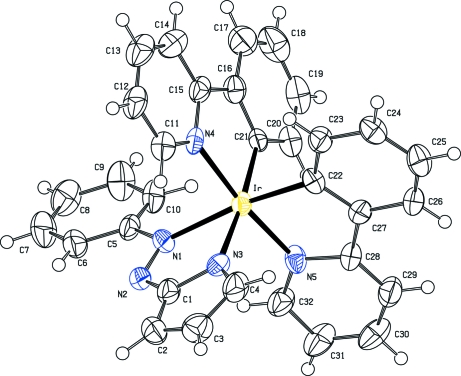
The structure of (I), showing 50% probability displacement ellipsoids and the atom-numbering scheme.

## supplementary crystallographic information

### Comment

Transition-metal (Ir) complexes that display intense phosphorescence from metal-to-ligand charge transfer (MLCT) excited states have been widely investigated (Baldo *et al.*, 2000; Pomestcheako *et al.*, 2003; Chen *et al.*, 2003). These Ir complexes with long excited-stated lifetimes and high luminescent efficiencies can be used in a variety of photonic applications such as photocatalysis and organic light-emitting diodes (Chin *et al.*, 1995). We report here the molecular structure of (I), (Fig. 1).

In the title compound (I), all the bond lengths and angles fall within normal ranges. Moreover, the Ir—C bond lengths are found to be shorter than the Ir—N bond lengths. In comparison with bis(2-pyridylphenyl)(acetylacetonate) iridium (Ir(ppy)2(acac)) which was reported in the literature (Lamansky *et al.*, 2001), this title complex displays longer Ir—N bond with the same *cis*-C, C *trans*-N, N chelate disposition. The dihedral angles between rings in the two ppy ligands are 5.2 (4)° [between rings N4/C11–C15 and C16–C21] and 5.5 (6)° [(between rings N5/C28–C32 and C22–C27].

### Experimental

To a stirring solution of 2-(2-phenylazo)-1*H*-pyrrole (60 mg,0.35 mmol) in dichloromethane (15 ml), sodium acetate (23 mg, 0.28 mmol) and [IrCl(ppy)2]2 (150 mg, 0.14 mmol) were added. The mixture was allowed to stir under an argon atmosphere at room temperature for 12 h. Then, the mixture was diluted with water and extracted thrice with 10 ml of dichloromethane. The organic extracts were combined and dried over anhydrous magnesium sulfate. After the solvent was removed *in vacuo*, the resulting residue was subjected to flash chromatography on silica gel using dichloromethane to afford the title compound (yield 75%). Red crystals of (I) suitable for X-ray structure analysis were grown from the mixture of dichloromethane and petroleum ether (*v*/*v*, 1:6).

### Refinement

All H-atoms were positioned geometrically and refined using a riding model with d(C—H) = 0.93 Å and *U*_iso_ = 1.2*U*_eq_ (C).

### Crystal data

**Table:**

[Ir(C_10_H_8_N_3_)(C_11_H_8_N)_2_]	*F*_000_ = 2624
*M**_r_* = 670.76	*D*_x_ = 1.729 Mg m^−^^3^
Monoclinic, *C*2/*c*	Mo *K*α radiation λ = 0.71073 Å
Hall symbol: -C 2yc	Cell parameters from 4482 reflections
*a* = 17.5606 (14) Å	θ = 4.6–52.7º
*b* = 11.0213 (9) Å	µ = 5.21 mm^−^^1^
*c* = 26.673 (2) Å	*T* = 293 (2) K
β = 93.2820 (10)º	Prism, red
*V* = 5153.9 (7) Å^3^	0.22 × 0.14 × 0.06 mm
*Z* = 8	

### Data collection

**Table:**

Bruker SMART APEX CCD area-detector diffractometer	5327 independent reflections
Radiation source: fine-focus sealed tube	4228 reflections with *I* > 2σ(*I*)
Monochromator: graphite	*R*_int_ = 0.140
*T* = 293(2) K	θ_max_ = 26.5º
φ and ω scans	θ_min_ = 2.2º
Absorption correction: multi-scan(SADABS; Sheldrick, 2001)	*h* = −21→22
*T*_min_ = 0.754, *T*_max_ = 1.000	*k* = −13→6
14220 measured reflections	*l* = −33→33

### Refinement

**Table:**

Refinement on *F*^2^	Secondary atom site location: difference Fourier map
Least-squares matrix: full	Hydrogen site location: inferred from neighbouring sites
*R*[*F*^2^ > 2σ(*F*^2^)] = 0.048	H-atom parameters constrained
*wR*(*F*^2^) = 0.107	*w* = 1/[σ^2^(*F*_o_^2^) + (0.0284*P*)^2^] where *P* = (*F*_o_^2^ + 2*F*_c_^2^)/3
*S* = 0.97	(Δ/σ)_max_ = 0.002
5327 reflections	Δρ_max_ = 4.15 e Å^−^^3^
343 parameters	Δρ_min_ = −2.37 e Å^−^^3^
Primary atom site location: structure-invariant direct methods	Extinction correction: none

### Special details

**Table:**

Geometry. All e.s.d.'s (except the e.s.d. in the dihedral angle between two l.s. planes) are estimated using the full covariance matrix. The cell e.s.d.'s are taken into account individually in the estimation of e.s.d.'s in distances, angles and torsion angles; correlations between e.s.d.'s in cell parameters are only used when they are defined by crystal symmetry. An approximate (isotropic) treatment of cell e.s.d.'s is used for estimating e.s.d.'s involving l.s. planes.
Refinement. Refinement of *F*^2^ against ALL reflections. The weighted *R*-factor *wR* and goodness of fit *S* are based on *F*^2^, conventional *R*-factors *R* are based on *F*, with *F* set to zero for negative *F*^2^. The threshold expression of *F*^2^ > σ(*F*^2^) is used only for calculating *R*-factors(gt) *etc*. and is not relevant to the choice of reflections for refinement. *R*-factors based on *F*^2^ are statistically about twice as large as those based on *F*, and *R*- factors based on ALL data will be even larger.

### Fractional atomic coordinates and isotropic or equivalent isotropic displacement parameters (Å^2^)

**Table:**

	*x*	*y*	*z*	*U*_iso_*/*U*_eq_	
Ir	0.118518 (14)	0.76861 (2)	0.384282 (9)	0.02997 (11)	
N1	0.0029 (3)	0.6954 (5)	0.3915 (2)	0.0339 (12)	
N2	−0.0012 (3)	0.5952 (5)	0.4165 (2)	0.0366 (13)	
N3	0.1331 (3)	0.6047 (5)	0.42223 (18)	0.0332 (12)	
N4	0.1050 (3)	0.8704 (5)	0.44743 (17)	0.0312 (12)	
N5	0.1403 (3)	0.6834 (5)	0.31918 (18)	0.0358 (12)	
C1	0.0655 (4)	0.5480 (6)	0.4316 (3)	0.0392 (16)	
C2	0.0800 (4)	0.4391 (6)	0.4596 (3)	0.0487 (19)	
H2	0.0442	0.3854	0.4712	0.058*	
C3	0.1589 (5)	0.4304 (7)	0.4660 (3)	0.052 (2)	
H3	0.1866	0.3689	0.4825	0.063*	
C4	0.1888 (4)	0.5342 (7)	0.4422 (3)	0.0485 (19)	
H4	0.2405	0.5509	0.4408	0.058*	
C5	−0.0713 (4)	0.7448 (6)	0.3781 (3)	0.0353 (16)	
C6	−0.1359 (4)	0.7030 (7)	0.3983 (3)	0.0497 (19)	
H6	−0.1327	0.6402	0.4216	0.060*	
C7	−0.2066 (5)	0.7536 (8)	0.3844 (4)	0.065 (3)	
H7	−0.2503	0.7227	0.3978	0.079*	
C8	−0.2124 (4)	0.8471 (8)	0.3516 (3)	0.060 (2)	
H8	−0.2597	0.8820	0.3434	0.072*	
C9	−0.1483 (4)	0.8905 (8)	0.3305 (3)	0.059 (2)	
H9	−0.1523	0.9549	0.3080	0.071*	
C10	−0.0775 (4)	0.8385 (7)	0.3426 (2)	0.0458 (18)	
H10	−0.0345	0.8658	0.3273	0.055*	
C11	0.1086 (4)	0.8273 (7)	0.4944 (2)	0.0412 (16)	
H11	0.1147	0.7442	0.4992	0.049*	
C12	0.1037 (4)	0.8995 (7)	0.5356 (2)	0.0483 (19)	
H12	0.1046	0.8655	0.5676	0.058*	
C13	0.0976 (4)	1.0214 (8)	0.5296 (3)	0.054 (2)	
H13	0.0958	1.0724	0.5573	0.065*	
C14	0.0939 (4)	1.0684 (7)	0.4814 (3)	0.0498 (19)	
H14	0.0887	1.1517	0.4766	0.060*	
C15	0.0978 (4)	0.9916 (6)	0.4402 (2)	0.0360 (15)	
C16	0.0931 (4)	1.0276 (6)	0.3870 (2)	0.0372 (15)	
C17	0.0809 (4)	1.1486 (7)	0.3711 (3)	0.054 (2)	
H17	0.0770	1.2102	0.3946	0.065*	
C18	0.0749 (5)	1.1744 (8)	0.3212 (4)	0.065 (2)	
H18	0.0661	1.2536	0.3103	0.078*	
C19	0.0820 (4)	1.0820 (8)	0.2866 (3)	0.055 (2)	
H19	0.0785	1.0997	0.2525	0.066*	
C20	0.0940 (4)	0.9656 (7)	0.3021 (3)	0.0435 (17)	
H20	0.0980	0.9050	0.2781	0.052*	
C21	0.1005 (3)	0.9341 (6)	0.3536 (2)	0.0328 (14)	
C22	0.2299 (4)	0.8077 (6)	0.3802 (2)	0.0330 (14)	
C23	0.2771 (4)	0.8709 (6)	0.4162 (2)	0.0388 (16)	
H23	0.2568	0.9012	0.4452	0.047*	
C24	0.3538 (4)	0.8875 (6)	0.4080 (3)	0.0417 (17)	
H24	0.3839	0.9310	0.4315	0.050*	
C25	0.3867 (4)	0.8421 (7)	0.3667 (3)	0.0475 (18)	
H25	0.4384	0.8534	0.3625	0.057*	
C26	0.3418 (4)	0.7792 (6)	0.3312 (3)	0.0440 (18)	
H26	0.3630	0.7492	0.3025	0.053*	
C27	0.2634 (4)	0.7606 (6)	0.3388 (3)	0.0361 (16)	
C28	0.2131 (4)	0.6904 (6)	0.3034 (2)	0.0350 (15)	
C29	0.2321 (4)	0.6370 (7)	0.2595 (3)	0.0487 (18)	
H29	0.2806	0.6485	0.2481	0.058*	
C30	0.1803 (5)	0.5667 (7)	0.2323 (3)	0.056 (2)	
H30	0.1938	0.5294	0.2029	0.067*	
C31	0.1093 (4)	0.5521 (8)	0.2487 (3)	0.055 (2)	
H31	0.0739	0.5027	0.2313	0.067*	
C32	0.0902 (4)	0.6121 (7)	0.2918 (3)	0.051 (2)	
H32	0.0410	0.6032	0.3026	0.061*	

### Atomic displacement parameters (Å^2^)

**Table:**

	*U*^11^	*U*^22^	*U*^33^	*U*^12^	*U*^13^	*U*^23^
Ir	0.02407 (15)	0.03860 (17)	0.02744 (14)	−0.00330 (11)	0.00326 (9)	−0.00461 (11)
N1	0.027 (3)	0.035 (3)	0.040 (3)	−0.008 (3)	0.004 (2)	−0.004 (3)
N2	0.031 (3)	0.035 (3)	0.044 (3)	−0.006 (3)	0.010 (2)	−0.009 (3)
N3	0.039 (3)	0.034 (3)	0.027 (3)	−0.001 (3)	0.006 (2)	−0.007 (2)
N4	0.021 (3)	0.048 (3)	0.025 (3)	−0.001 (3)	0.006 (2)	−0.006 (2)
N5	0.029 (3)	0.052 (3)	0.027 (3)	−0.009 (3)	0.002 (2)	−0.002 (3)
C1	0.028 (4)	0.044 (4)	0.046 (4)	−0.001 (3)	0.006 (3)	−0.009 (3)
C2	0.046 (5)	0.041 (4)	0.060 (5)	−0.003 (4)	0.014 (4)	0.007 (4)
C3	0.053 (5)	0.047 (4)	0.057 (5)	0.010 (4)	0.001 (4)	0.004 (4)
C4	0.030 (4)	0.056 (5)	0.060 (5)	0.008 (4)	0.004 (3)	−0.005 (4)
C5	0.024 (4)	0.044 (4)	0.038 (4)	0.001 (3)	0.006 (3)	−0.009 (3)
C6	0.039 (4)	0.043 (4)	0.068 (5)	0.000 (4)	0.016 (4)	0.005 (4)
C7	0.027 (4)	0.079 (6)	0.091 (7)	−0.004 (4)	0.012 (4)	−0.001 (5)
C8	0.038 (4)	0.080 (6)	0.062 (5)	0.014 (5)	−0.003 (4)	−0.005 (5)
C9	0.040 (4)	0.088 (6)	0.049 (5)	0.007 (5)	−0.001 (4)	0.017 (4)
C10	0.029 (4)	0.068 (5)	0.041 (4)	0.002 (4)	0.006 (3)	0.001 (4)
C11	0.036 (4)	0.051 (4)	0.037 (4)	0.001 (4)	0.003 (3)	0.001 (3)
C12	0.044 (4)	0.074 (6)	0.027 (4)	−0.002 (4)	0.006 (3)	−0.011 (4)
C13	0.047 (5)	0.077 (6)	0.038 (4)	0.000 (4)	0.000 (3)	−0.023 (4)
C14	0.047 (5)	0.052 (5)	0.050 (4)	0.007 (4)	0.001 (4)	−0.011 (4)
C15	0.024 (3)	0.046 (4)	0.039 (4)	0.002 (3)	0.004 (3)	−0.007 (3)
C16	0.028 (3)	0.045 (4)	0.039 (4)	0.002 (3)	0.003 (3)	0.004 (3)
C17	0.048 (5)	0.049 (5)	0.067 (5)	0.003 (4)	0.007 (4)	0.005 (4)
C18	0.047 (5)	0.058 (5)	0.091 (7)	0.005 (5)	0.004 (5)	0.032 (5)
C19	0.037 (4)	0.077 (6)	0.052 (5)	−0.001 (4)	0.007 (3)	0.021 (4)
C20	0.035 (4)	0.056 (5)	0.039 (4)	−0.006 (4)	0.005 (3)	0.005 (3)
C21	0.026 (3)	0.029 (3)	0.045 (4)	−0.002 (3)	0.013 (3)	0.003 (3)
C22	0.030 (4)	0.036 (3)	0.033 (3)	−0.004 (3)	0.001 (3)	0.007 (3)
C23	0.030 (4)	0.047 (4)	0.040 (4)	−0.004 (3)	0.001 (3)	−0.008 (3)
C24	0.030 (4)	0.046 (4)	0.048 (4)	−0.005 (3)	−0.008 (3)	0.004 (3)
C25	0.021 (3)	0.062 (5)	0.059 (5)	−0.003 (4)	−0.001 (3)	0.003 (4)
C26	0.032 (4)	0.048 (4)	0.053 (4)	0.001 (3)	0.016 (3)	−0.004 (3)
C27	0.033 (4)	0.035 (4)	0.041 (4)	0.002 (3)	0.006 (3)	−0.003 (3)
C28	0.029 (3)	0.046 (4)	0.031 (3)	0.004 (3)	0.006 (3)	0.002 (3)
C29	0.046 (4)	0.058 (5)	0.044 (4)	0.002 (4)	0.015 (3)	−0.005 (4)
C30	0.059 (5)	0.068 (5)	0.040 (4)	0.018 (5)	−0.002 (4)	−0.020 (4)
C31	0.045 (5)	0.082 (6)	0.038 (4)	0.007 (4)	−0.010 (3)	−0.029 (4)
C32	0.037 (4)	0.063 (5)	0.054 (5)	−0.003 (4)	0.004 (3)	−0.025 (4)

### Geometric parameters (Å, °)

**Table:**

Ir—C22	2.012 (6)	C12—H12	0.9300
Ir—C21	2.016 (6)	C13—C14	1.383 (10)
Ir—N5	2.030 (5)	C13—H13	0.9300
Ir—N4	2.049 (5)	C14—C15	1.394 (9)
Ir—N3	2.079 (5)	C14—H14	0.9300
Ir—N1	2.204 (5)	C15—C16	1.469 (9)
N1—N2	1.293 (7)	C16—C21	1.374 (9)
N1—C5	1.438 (9)	C16—C17	1.411 (10)
N2—C1	1.324 (8)	C17—C18	1.361 (11)
N3—C4	1.336 (8)	C17—H17	0.9300
N3—C1	1.377 (8)	C18—C19	1.384 (12)
N4—C11	1.339 (8)	C18—H18	0.9300
N4—C15	1.355 (9)	C19—C20	1.360 (10)
N5—C32	1.362 (8)	C19—H19	0.9300
N5—C28	1.370 (8)	C20—C21	1.417 (9)
C1—C2	1.429 (9)	C20—H20	0.9300
C2—C3	1.389 (11)	C22—C27	1.383 (10)
C2—H2	0.9300	C22—C23	1.415 (8)
C3—C4	1.423 (10)	C23—C24	1.389 (9)
C3—H3	0.9300	C23—H23	0.9300
C4—H4	0.9300	C24—C25	1.368 (9)
C5—C6	1.364 (10)	C24—H24	0.9300
C5—C10	1.400 (10)	C25—C26	1.382 (10)
C6—C7	1.392 (11)	C25—H25	0.9300
C6—H6	0.9300	C26—C27	1.418 (11)
C7—C8	1.352 (12)	C26—H26	0.9300
C7—H7	0.9300	C27—C28	1.474 (9)
C8—C9	1.373 (11)	C28—C29	1.369 (9)
C8—H8	0.9300	C29—C30	1.371 (10)
C9—C10	1.390 (9)	C29—H29	0.9300
C9—H9	0.9300	C30—C31	1.355 (11)
C10—H10	0.9300	C30—H30	0.9300
C11—C12	1.364 (9)	C31—C32	1.384 (9)
C11—H11	0.9300	C31—H31	0.9300
C12—C13	1.357 (11)	C32—H32	0.9300
			
C22—Ir—C21	85.1 (2)	C13—C12—H12	120.3
C22—Ir—N5	79.7 (2)	C11—C12—H12	120.3
C21—Ir—N5	96.0 (2)	C12—C13—C14	118.7 (7)
C22—Ir—N4	94.9 (2)	C12—C13—H13	120.6
C21—Ir—N4	79.4 (2)	C14—C13—H13	120.6
N5—Ir—N4	173.2 (2)	C13—C14—C15	120.3 (7)
C22—Ir—N3	96.8 (2)	C13—C14—H14	119.9
C21—Ir—N3	174.5 (2)	C15—C14—H14	119.9
N5—Ir—N3	89.5 (2)	N4—C15—C14	119.6 (6)
N4—Ir—N3	95.3 (2)	N4—C15—C16	113.8 (6)
C22—Ir—N1	170.6 (2)	C14—C15—C16	126.5 (7)
C21—Ir—N1	104.0 (2)	C21—C16—C17	122.1 (6)
N5—Ir—N1	97.2 (2)	C21—C16—C15	114.9 (6)
N4—Ir—N1	88.74 (19)	C17—C16—C15	123.0 (7)
N3—Ir—N1	74.2 (2)	C18—C17—C16	119.6 (8)
N2—N1—C5	112.0 (5)	C18—C17—H17	120.2
N2—N1—Ir	115.9 (4)	C16—C17—H17	120.2
C5—N1—Ir	131.7 (4)	C17—C18—C19	119.6 (8)
N1—N2—C1	114.7 (6)	C17—C18—H18	120.2
C4—N3—C1	106.4 (6)	C19—C18—H18	120.2
C4—N3—Ir	140.1 (5)	C20—C19—C18	120.7 (7)
C1—N3—Ir	113.5 (4)	C20—C19—H19	119.6
C11—N4—C15	118.9 (6)	C18—C19—H19	119.6
C11—N4—Ir	125.1 (5)	C19—C20—C21	121.8 (7)
C15—N4—Ir	115.9 (4)	C19—C20—H20	119.1
C32—N5—C28	117.0 (6)	C21—C20—H20	119.1
C32—N5—Ir	125.3 (5)	C16—C21—C20	116.1 (6)
C28—N5—Ir	117.5 (4)	C16—C21—Ir	115.7 (5)
N2—C1—N3	121.5 (6)	C20—C21—Ir	128.1 (5)
N2—C1—C2	128.1 (7)	C27—C22—C23	117.9 (6)
N3—C1—C2	110.3 (6)	C27—C22—Ir	115.0 (5)
C3—C2—C1	105.6 (7)	C23—C22—Ir	127.0 (5)
C3—C2—H2	127.2	C24—C23—C22	119.6 (6)
C1—C2—H2	127.2	C24—C23—H23	120.2
C2—C3—C4	106.3 (6)	C22—C23—H23	120.2
C2—C3—H3	126.9	C25—C24—C23	122.4 (6)
C4—C3—H3	126.9	C25—C24—H24	118.8
N3—C4—C3	111.4 (6)	C23—C24—H24	118.8
N3—C4—H4	124.3	C24—C25—C26	119.0 (6)
C3—C4—H4	124.3	C24—C25—H25	120.5
C6—C5—C10	118.8 (7)	C26—C25—H25	120.5
C6—C5—N1	122.3 (6)	C25—C26—C27	119.6 (7)
C10—C5—N1	118.8 (6)	C25—C26—H26	120.2
C5—C6—C7	120.5 (8)	C27—C26—H26	120.2
C5—C6—H6	119.8	C22—C27—C26	121.4 (6)
C7—C6—H6	119.8	C22—C27—C28	116.2 (6)
C8—C7—C6	120.8 (9)	C26—C27—C28	122.4 (7)
C8—C7—H7	119.6	N5—C28—C29	121.0 (6)
C6—C7—H7	119.6	N5—C28—C27	111.6 (6)
C7—C8—C9	119.8 (8)	C29—C28—C27	127.3 (7)
C7—C8—H8	120.1	C28—C29—C30	120.6 (7)
C9—C8—H8	120.1	C28—C29—H29	119.7
C8—C9—C10	120.3 (8)	C30—C29—H29	119.7
C8—C9—H9	119.9	C31—C30—C29	119.5 (7)
C10—C9—H9	119.9	C31—C30—H30	120.3
C9—C10—C5	119.7 (7)	C29—C30—H30	120.3
C9—C10—H10	120.1	C30—C31—C32	118.9 (7)
C5—C10—H10	120.1	C30—C31—H31	120.6
N4—C11—C12	123.1 (7)	C32—C31—H31	120.6
N4—C11—H11	118.5	N5—C32—C31	122.7 (7)
C12—C11—H11	118.5	N5—C32—H32	118.6
C13—C12—C11	119.4 (7)	C31—C32—H32	118.6
			
C21—Ir—N1—N2	−171.3 (4)	C11—N4—C15—C14	−0.4 (9)
N5—Ir—N1—N2	90.7 (4)	Ir—N4—C15—C14	−175.4 (5)
N4—Ir—N1—N2	−92.6 (4)	C11—N4—C15—C16	−178.9 (6)
N3—Ir—N1—N2	3.3 (4)	Ir—N4—C15—C16	6.0 (7)
C21—Ir—N1—C5	1.0 (6)	C13—C14—C15—N4	0.2 (11)
N5—Ir—N1—C5	−97.1 (6)	C13—C14—C15—C16	178.6 (7)
N4—Ir—N1—C5	79.7 (6)	N4—C15—C16—C21	−3.1 (9)
N3—Ir—N1—C5	175.5 (6)	C14—C15—C16—C21	178.4 (7)
C5—N1—N2—C1	−177.5 (5)	N4—C15—C16—C17	176.2 (6)
Ir—N1—N2—C1	−3.7 (7)	C14—C15—C16—C17	−2.3 (12)
C22—Ir—N3—C4	2.4 (7)	C21—C16—C17—C18	1.2 (12)
N5—Ir—N3—C4	82.0 (7)	C15—C16—C17—C18	−178.1 (7)
N4—Ir—N3—C4	−93.2 (7)	C16—C17—C18—C19	−1.0 (12)
N1—Ir—N3—C4	179.6 (7)	C17—C18—C19—C20	0.8 (12)
C22—Ir—N3—C1	−179.4 (4)	C18—C19—C20—C21	−0.7 (12)
N5—Ir—N3—C1	−99.8 (4)	C17—C16—C21—C20	−1.0 (10)
N4—Ir—N3—C1	85.0 (4)	C15—C16—C21—C20	178.3 (6)
N1—Ir—N3—C1	−2.2 (4)	C17—C16—C21—Ir	179.4 (6)
C22—Ir—N4—C11	−95.9 (5)	C15—C16—C21—Ir	−1.3 (8)
C21—Ir—N4—C11	−180.0 (5)	C19—C20—C21—C16	0.8 (10)
N3—Ir—N4—C11	1.5 (5)	C19—C20—C21—Ir	−179.7 (5)
N1—Ir—N4—C11	75.5 (5)	C22—Ir—C21—C16	−92.5 (5)
C22—Ir—N4—C15	78.9 (5)	N5—Ir—C21—C16	−171.6 (5)
C21—Ir—N4—C15	−5.2 (4)	N4—Ir—C21—C16	3.4 (5)
N3—Ir—N4—C15	176.2 (4)	N1—Ir—C21—C16	89.4 (5)
N1—Ir—N4—C15	−109.8 (5)	C22—Ir—C21—C20	88.0 (6)
C22—Ir—N5—C32	174.5 (6)	N5—Ir—C21—C20	8.9 (6)
C21—Ir—N5—C32	−101.6 (6)	N4—Ir—C21—C20	−176.1 (6)
N3—Ir—N5—C32	77.4 (6)	N1—Ir—C21—C20	−90.1 (6)
N1—Ir—N5—C32	3.4 (6)	C21—Ir—C22—C27	−97.3 (5)
C22—Ir—N5—C28	−0.1 (5)	N5—Ir—C22—C27	−0.3 (5)
C21—Ir—N5—C28	83.8 (5)	N4—Ir—C22—C27	−176.2 (5)
N3—Ir—N5—C28	−97.2 (5)	N3—Ir—C22—C27	87.9 (5)
N1—Ir—N5—C28	−171.2 (5)	C21—Ir—C22—C23	86.6 (6)
N1—N2—C1—N3	1.8 (9)	N5—Ir—C22—C23	−176.4 (6)
N1—N2—C1—C2	−179.9 (6)	N4—Ir—C22—C23	7.8 (6)
C4—N3—C1—N2	179.9 (6)	N3—Ir—C22—C23	−88.2 (6)
Ir—N3—C1—N2	1.1 (8)	C27—C22—C23—C24	2.4 (10)
C4—N3—C1—C2	1.4 (7)	Ir—C22—C23—C24	178.3 (5)
Ir—N3—C1—C2	−177.4 (4)	C22—C23—C24—C25	−1.7 (11)
N2—C1—C2—C3	−179.7 (7)	C23—C24—C25—C26	1.1 (11)
N3—C1—C2—C3	−1.3 (8)	C24—C25—C26—C27	−1.4 (11)
C1—C2—C3—C4	0.6 (8)	C23—C22—C27—C26	−2.7 (10)
C1—N3—C4—C3	−1.0 (8)	Ir—C22—C27—C26	−179.1 (5)
Ir—N3—C4—C3	177.3 (5)	C23—C22—C27—C28	177.1 (6)
C2—C3—C4—N3	0.2 (8)	Ir—C22—C27—C28	0.7 (8)
N2—N1—C5—C6	12.3 (9)	C25—C26—C27—C22	2.2 (11)
Ir—N1—C5—C6	−160.2 (5)	C25—C26—C27—C28	−177.6 (7)
N2—N1—C5—C10	−167.2 (6)	C32—N5—C28—C29	6.0 (10)
Ir—N1—C5—C10	20.3 (9)	Ir—N5—C28—C29	−179.0 (5)
C10—C5—C6—C7	−0.7 (12)	C32—N5—C28—C27	−174.5 (6)
N1—C5—C6—C7	179.8 (7)	Ir—N5—C28—C27	0.5 (8)
C5—C6—C7—C8	−1.8 (14)	C22—C27—C28—N5	−0.8 (9)
C6—C7—C8—C9	2.1 (14)	C26—C27—C28—N5	179.0 (6)
C7—C8—C9—C10	0.1 (13)	C22—C27—C28—C29	178.7 (7)
C8—C9—C10—C5	−2.6 (12)	C26—C27—C28—C29	−1.5 (11)
C6—C5—C10—C9	2.8 (11)	N5—C28—C29—C30	−5.4 (11)
N1—C5—C10—C9	−177.7 (6)	C27—C28—C29—C30	175.2 (7)
C15—N4—C11—C12	1.5 (10)	C28—C29—C30—C31	1.2 (12)
Ir—N4—C11—C12	176.0 (5)	C29—C30—C31—C32	2.1 (13)
N4—C11—C12—C13	−2.4 (11)	C28—N5—C32—C31	−2.6 (11)
C11—C12—C13—C14	2.1 (11)	Ir—N5—C32—C31	−177.2 (6)
C12—C13—C14—C15	−1.1 (12)	C30—C31—C32—N5	−1.4 (13)
